# Acetyl-Salicylic Acid in the Treatment of Chorea

**Published:** 1907-07-27

**Authors:** John Allan


### The General Practitioner's Column.

[Contributions to this Column are invited, and if accepted will be paid for.]

### ACETYL-SALICYLIC ACID IN THE TREATMENT OF CHOREA.

By JOHN ALLAN, M.B., Ch.B.

Chorea is a disease which is frequently met with in general practice, and although a number of drugs have been employed with varying success in its treatment, the condition often baffles the resources of the medical attendant. Personally I have tried four drugs—namely, antipyrin, salicylate of sodium, arsenic, and acetyl-salicylic acid. I have never been able to satisfy myself that much good resulted from the first two of these. With arsenic and acetyl-salicylic acid relief of the condition occurs so quickly as to justify one in the belief that the favourable issue results from their employment. When one considers that many cases of chorea are undoubtedly associated with a rheumatic diathesis, the comparative failure of salicylate of sodium is remarkable, whereas acetyl-salicylic acid appears to be a much more reliable remedy.

### An Illustrative Case.

The following three cases have been treated with the latter drug:—

Case 1.—E. B., a little girl seven years of age, was first seen on January 16, 1907. She was suffering from acute chorea, which began on January 14. The movements were violent, and she was quite unable to feed herself. She was wriggling, twisting, shrugging, and grimacing continuously, except for a few hours during the night when she slept. There was no history of previous rheumatic fever, but both the father and the mother had suffered from rheumatism. The cardiac sounds were natural. She was put to bed, and the following mixture was prescribed:—

| | | |
|---|---|---|
| ℞ | Acetyl-salicylic acid ... ... ... | ʒiiss. |
| | Gum acacia ... ... ... ... ... | q.s. |
| | Syr. aurantii ... ... ... ... | ʒj. |
| | Aq. ad ... ... ... ... ... | ʒiv. |
| Sig. | Two teaspoonfuls every six hours. | |

Very soon the movements became less marked, and at the end of a week she was much better. The mixture was now given thrice daily. At the end of the second week the movement had ceased. The medicine was then stopped, and the patient was allowed to get up. She continued to do well, and the attack was considered cured less than three weeks after the onset.

### Two Other Cases.

Case 2.—R. H., a girl nine years of age, was seen on January 18, 1907, on account of an attack of subacute chorea. The child had always been "nervous," but she had not previously had chorea. She had had rheumatic fever towards the end of 1904. The movements were well marked, but not violent, and had been noticed for about a fortnight. She could feed herself with difficulty, but she was quite unable to perform more delicate movements, such as fastening the buttons of her dress, threading a needle, etc. Over the mitral area a systolic bruit could be heard, propagated towards the angle of the scapula. She was put on the above mixture, except that 12½ grains of acetyl-salicylic acid were given in each dose. Rapid improvement followed, and in about three weeks she was cured of the attack.

Case 3.—C. M., a boy eleven years of age, came under observation on March 30, 1907. His case was very chronic, and he had been more or less affected since October 1906. This was his second attack, the first one having occurred some three years previously. There was no history of previous rheumatic fever or "growing pains," and, so far as could be ascertained, there was no evidence of any rheumatic tendency existing in the family. The choreiform movements were slight, but were always present when he was awake. The cardiac system was healthy, the heart sounds being quite clear and perfectly normal. He was given fifteen grains of acetyl-salicylic acid four times a day, and this was reduced to thrice daily in ten days' time. In three weeks' time the movements had ceased, and he was then given arsenic in increasing doses, as he was very anæmic. Twelve minims of liquor arsenicalis three times a day was the maximum dose given. He was discharged cured on May 1, 1907.

### Some Points in the Treatment of Chorea.

No mention has been made of certain important points which must be attended to in the treatment of chorea.

It is practically essential that all cases should be treated by rest in bed, otherwise drugs will be of little avail. Many cases require strict isolation, and the patients should have plenty of milk to drink. A considerable percentage of cases can be eventually cured by rest in bed, etc., without the aid of drugs; but there can be no doubt that certain drugs tend to bring about more rapid recovery.

My own experience has been that acetyl-salicylic acid offers the best means of obtaining this happy result. To get good results one must push the drug and give large doses. It can be given in powder, but for children a mixture such as the above is preferable. The gum acacia simply serves to hold the drug in suspension, and the syrup of orange is an agreeable flavouring agent. Children take it well in such a mixture, and it is not so objectionable as salicylate of soda, the sweet mawkish taste of which is extremely difficult to cover. The cases selected illustrate chorea in its acute, subacute, and chronic forms, and it is to be noted that each form responded equally well to the drug.

